# Targeted delivery of irinotecan to colon cancer cells using epidermal growth factor receptor-conjugated liposomes

**DOI:** 10.1186/s12938-022-01012-8

**Published:** 2022-08-02

**Authors:** Yongwei Liu, Xinghui Li, Renqun Pen, Wei Zuo, Ya Chen, Xiuying Sun, Juhua Gou, Qianwen Guo, Maoling Wen, Wuqi Li, Shuangjiang Yu, Hao Liu, Min Huang

**Affiliations:** 1 Department of Infection, Rongchang District People’s Hospital of Chongqing, No.11 Changyuan St Square North Rd, Rongchang District, Chongqing, 402460 China; 2 Department of Digestion, Rongchang District People’s Hospital of Chongqing, No.11 Changyuan St Square North Rd, Rongchang District, Chongqing, 402460 China; 3 Department of Neurosurgery, The First Hospital Affiliated to Army Military Medical University (Southwest Hospital), Chongqing, 400038 China; 4 Department of Digestion, The Affiliated Hospital of North Sichuan Medical College, No.1, MaoYuan South Rd, Shunqing District, Nanchong, 637000 Sichuan China

**Keywords:** Irinotecan (CPT-11), DSPE-PEG_2000_, EGFR, Colorectal cancer, SW620 cell

## Abstract

**Background:**

CPT-11 (irinotecan) is one of the most efficient agents used for colorectal cancer chemotherapy. However, as for many other chemotherapeutic drugs, how to minimize the side effects of CPT-11 still needs to be thoroughly described.

**Objectives:**

This study aimed to develop the CPT-11-loaded DSPE-PEG 2000 targeting EGFR liposomal delivery system and characterize its targeting specificity and therapeutic effect on colorectal cancer (CRC) cells in vitro and in vivo.

**Results:**

The synthesized liposome exhibited spherical shapes (84.6 ± 1.2 nm to 150.4 nm ± 0.8 nm of estimated average sizes), good stability, sustained release, and enough drug loading (55.19%). For in vitro experiments, SW620 cells treated with CPT-11-loaded DSPE-PEG_2000_ targeting EGFR liposome showed lower survival extended level of intracellular ROS production. In addition, it generated an enhanced apoptotic cell rate by upregulating the protein expression of both cleaved-caspase-3 and cleaved-caspase-9 compared with those of SW620 cells treated with free CPT-11. Importantly, the xenograft model showed that both the non-target and EGFR-targeted liposomes significantly inhibited tumor growth compared to free CPT-11.

**Conclusions:**

Compared with the non-target CPT-11-loaded DSPE-PEG_2000_ liposome, CPT-11-loaded DSPE-PEG2000 targeting EGFR liposome treatment showed much better antitumor activity in vitro in vivo. Thus, our findings provide new assets and expectations for CRC targeting therapy.

**Supplementary Information:**

The online version contains supplementary material available at 10.1186/s12938-022-01012-8.

## Introduction

Colorectal cancer (CRC) represents one of the most prevalent malignant tumors worldwide. Although several therapeutic strategies toward colorectal cancer have been widely reported during the last decade, its poor prognosis after lymphatic metastasis or distant organs remains the principal cause of high mortality and low overall 5-year survival rate [1, 2]. Except for surgery, first-line chemotherapeutic drugs for CRC, such as 5-fluorouracil, irinotecan, and oxaliplatin, are the most used drugs for routine treatments or following surgery [3]. Long-term and systematic application of chemotherapeutic drugs generates dose-dependent toxicity on untargeted cells, tissues, and organs. Oral administration is generally recommended, even though it results in a short circulation half-life and severe side effects, which significantly reduce the efficiency of many first-line anticancer reagents and lead to chemotherapy failures [4].

Increasing numbers of reports have previously highlighted effective and promising features for drugs delivery through nano-carriers like liposomes and nanoparticles [5, 6]. Liposomes are the lipid bilayers composed of phospholipids and cholesterol similar to the cell membrane with high biocompatibility [7]. They are easily stored in tumor tissues and can be easily targeted by specific antibodies [8]. Recent studies have demonstrated that liposomes loaded with antitumor reagents like doxorubicin and docetaxel promoted tumor uptake and lowered systemic toxicity [9].

Epidermal growth factor receptor (EGFR) is among the most prominent targets in colorectal cancers (CRCs) treatments. The EGFR-targeted nanoparticles delivery effectively enhanced the inhibition of colorectal cancer growth in a mouse model, suggesting EGFR as a promising target for liposomal drug delivery systems [10, 11]. Interestingly, the concentration of CPT-11, mainly used for CRC treatment, was reported to be highly enhanced in the glioblastoma xenograft tumors cells treated with CPT-11 loaded liposome [12]. In addition, SN-38 proteins, the active metabolites of irinotecan, were less expressed in colon and liver cancer cells [13, 14], while SN-38-loaded targeted liposome induced a much lower IC50, continuous release of SN38 than the non-liposomal SN38 [15]. Unfortunately, SN38 exerts its anticancer effects by blocking the DNA synthesis [16]; it also produces severe side effects like vomiting, myelosuppression, nausea, and diarrhea [17]. Nowadays, the utilization of nanoparticles for drugs delivery to various tumor tissues represents a promising method for cancer chemotherapy. Yet, to our knowledge, this is the first report on EGFR-targeted liposomes as CPT-11 delivery system for cancer therapy.

Our study aimed to prepare EGFR-targeted DSPE-PEG2000 liposomes as CPT-11 delivery systems for colorectal cancer therapy. It evaluated the newly developed drug delivery system’s systemic toxicity, drug release, and antitumor efficacy. The delivery of CPT-11-loaded DSPE-PEG 2000 targeting EGFR liposome to colon cancer cells enhanced the antitumor activity of CPT-11 in SW620 cells in vitro. Thus, EGFR-targeted DSPE-PEG2000 liposomes as CPT-11 delivery systems could be a promising road for CRC treatment.

## Results

### Characterization of CPT-11-loaded DSPE-PEG_2000_ liposome

**Fig. 1:**
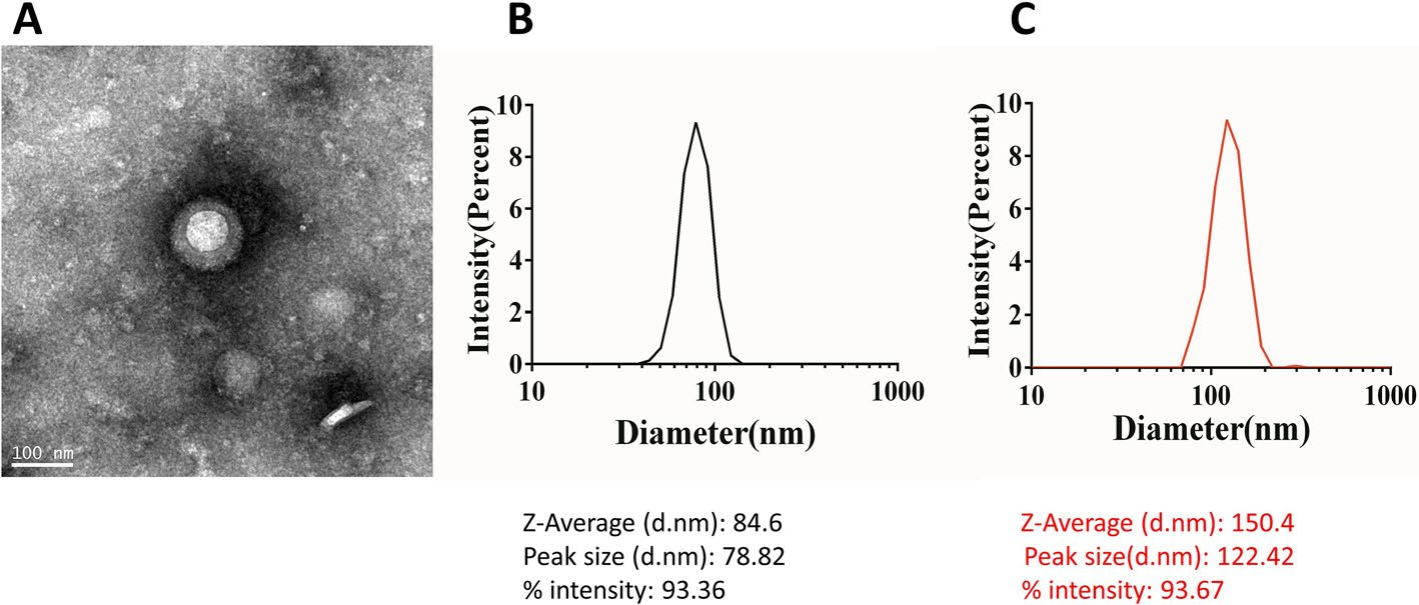
The morphology and particle size of CPT-11-loaded DSPE-PEG_2000_ liposome. **A** The morphology of CPT-11-loaded DSPE-PEG_2000_ targeting EGFR liposome; **B** CPT-11-loaded DSPE-PEG_2000_ liposome; **C** the particle size of CPT-11-loaded DSPE-PEG_2000_ liposome targeting EGFR. CPT-11-Lipo: CPT-11-loaded DSPE-PEG_2000_ liposome; EGFR-Lipo-CPT-11: CPT-11-loaded DSPE-PEG_2000_ liposome coated with anti-epidermal growth factor receptor (EGFR) antibody

The CPT-11-loaded DSPE-PEG_2000_ liposome (Lipo-CPT-11) was synthesized by a self-assembly method as described in the previous studies [18]. Subsequently, the monoclonal antibody of EGFR-targeted was coupled with CPT-11-loaded DSPE-PEG_2000_ liposome to form the complex of CPT-11-loaded DSPE-PEG_2000_ targeting EGFR liposome (EGFR-Lipo-CPT-11). The EGFR-targeted liposome exhibited spherical or irregular shapes through TEM (Fig. 1A). Before preparing the CPT-11-loaded liposome, the average size of particle commercialized DSPE-PEG_2000_-NH_2_ was about 75 nm in diameter. After assembly with CPT-11, the particle sizes of CPT-11-loaded DSPE-PEG_2000_ liposomes displayed an increased average size of 84.6 ± 1.2 nm in diameter (Fig. 1B), indicating that the liposomal nanocarriers successfully loaded the irinotecan to form CPT-11-loaded DSPE-PEG 2000. After that, the EGFR antibody was coupled to the PBS solution, and the characterizations of the CPT-11-loaded DSPE-PEG 2000 targeting EGFR liposome were further evaluated. Finally, the average sizes of EGFR-coated CPT-11-loaded DSPE-PEG2000 liposomes were about 150.4 nm ± 0.8 nm in diameter (Fig. 1C), suggesting the successful coating of EGFR.

### Physical stability, cumulative release, and drug loading of CPT-11-loaded DSPE-PEG_2000_ targeting EGFR liposome in vitro

**Fig. 2:**
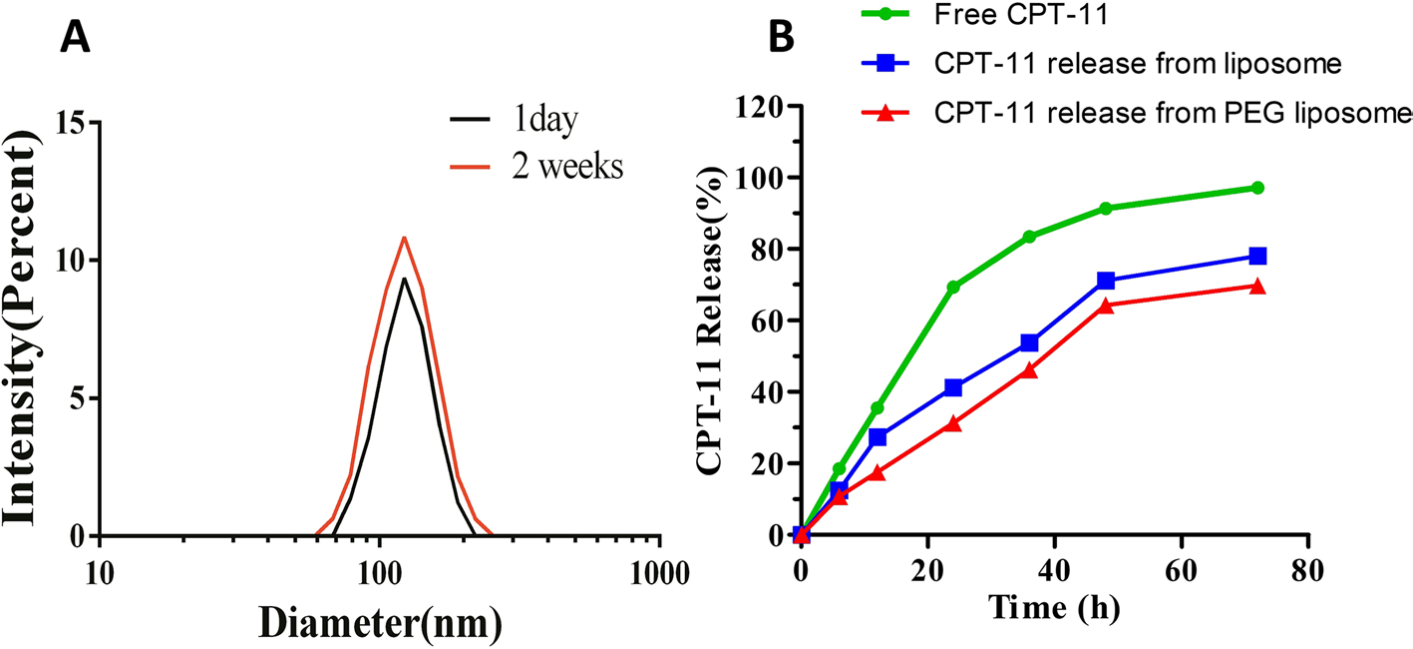
The physical stability and cumulative release profile of CPT-11-loaded DSPE-PEG_2000_ liposome in vitro. **A** The physical strength was measured by the particle sizes of CPT-11-loaded DSPE-PEG_2000_ liposome at the 1st day and 14th day after preparation; **B** the cumulative release profile of the prepared liposome

To evaluate the physical stability of EGFR-Lipo-CPT-11, we investigated both the liposome expansion and the drug release rate at the 1st and 14th days after assembly. The results in Fig. 2A showed how the particle size on the 1st day was almost consistent with that of the 14th day, indicating that EGFR-Lipo-CPT-11 was very stable. In addition, to explore the efficiency of the EGFR-Lipo-CPT-11 drug delivery system, we prepared three groups, including the free irinotecan as control, irinotecan in standard liposomes, and irinotecan in PEG-coated liposomes. In comparison with the control (passive diffusion of free CPT-11), the in vitro release of CPT-11-loaded DSPE-PEG2000 targeting EGFR liposome evaluated in PBS (pH 7.4) solution at 37 °C exhibited a sustained drug release, with only about 35% of the irinotecan released at the first 12th h. The free CPT-11 was released at the fastest rate, while PEG liposomes released the CPT-11 at a slower rate than standard liposomes (Fig. 2B). The concentration of irinotecan in the liposomal nanoparticles was 0.4213 mg/mL. The drug loading rate was (0.4213 * 2.62/2) * 100 = 55.19% and the release rate of the liposomal nanoparticles was detected at 6 h and 12 h after dialysis. The concentration of irinotecan was detected at 24 h, 36 h, 48 h, 72 h, and the release rate gradually reached more than 95% after 72 h. Our results suggested that using the PEG-loaded liposomes might reduce the release rate of CPT-11 and improve the drug stability.

### In vitro anticancer effects of EGFR-Lipo-CPT-11

**Fig. 3:**
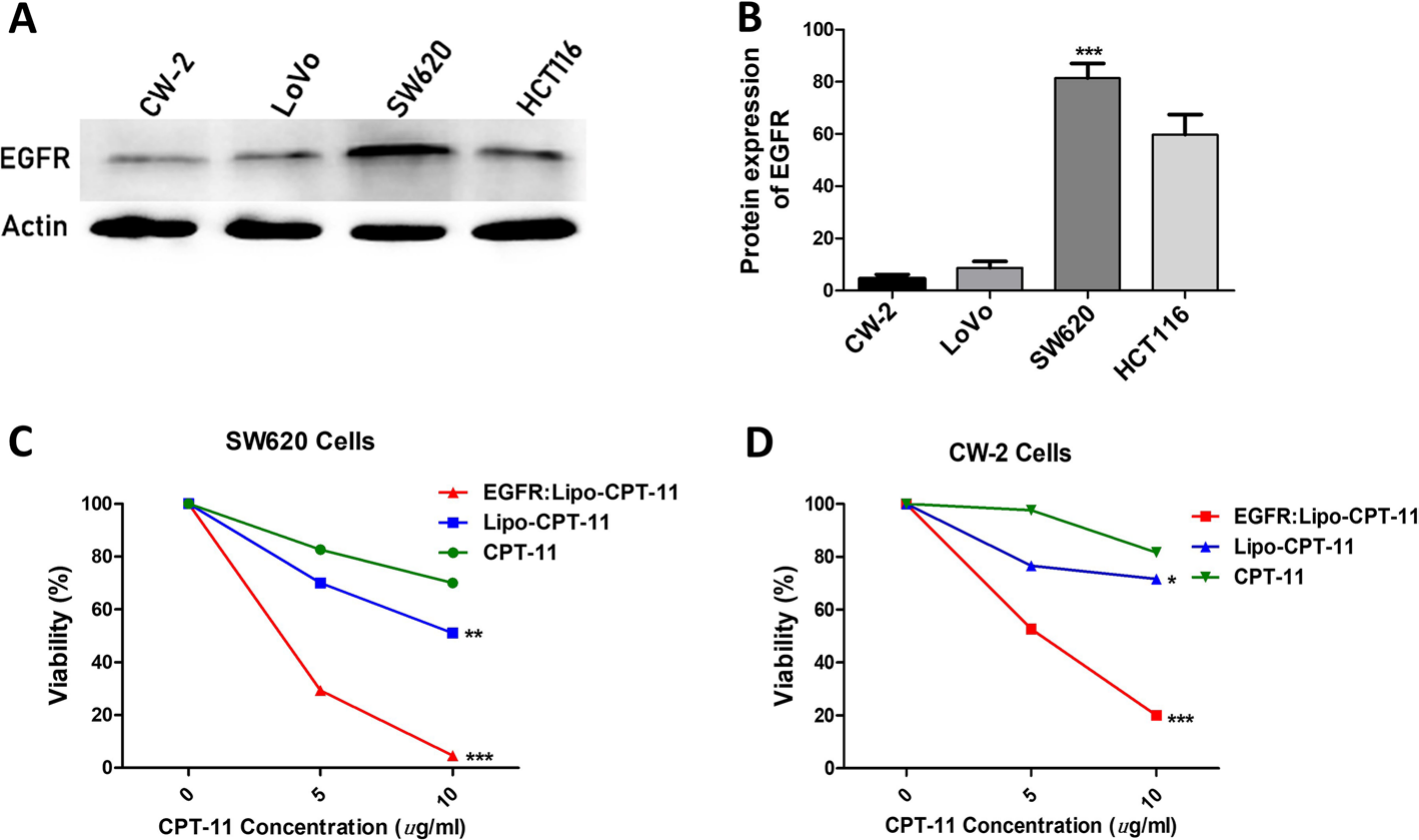
In vitro anticancer effects. SW620 and CW-2 cell lines expressed the highest and lowest levels of EGFR (**A** and** B**). Under treatments with free CPT-11, liposomal CPT-11 (Lipo-CPT-11) or EGFR-Lipo-CPT-11 for 24 h, SW620 cells [highest EGFR-expressing cell lines] showed a significant reduction of cell viability while the CW-2 cell lines (lowest EGFR-expressing cell lines) were less affected (**C** and **D**)

To demonstrate that the CPT11-loaded liposome targets EGFR overexpressed tumor cells, we performed western blotting analysis on four different CRC cell lines, including CW-2, LoVo, SW620 and CT116 cells. SW620 and CW-2 cells, which expressed EGFR at the highest and lowest levels, respectively, were used to test the targeting potential of EGFR-Lipo-CPT-11 in EGFR-expressing cancer cells (Fig. 3A and B). When EGFR-Lipo-CPT-11 was used instead of Lipo-CPT-11, the viability of SW620 cell lines displayed the lowest rate; thus, the cytotoxicity of EGFR-Lipo-CPT-11 against SW620 cells was significantly more significant than Lipo-CPT-11 at 10 g/mL. At the same time, the vitality of CW-2 cells that barely expressed EGFR was unaffected by the EGFR-Lipo-CPT-11 and Lipo-CPT-11 treatments (Fig. 3C and D). These findings suggested that EGFR-Lipo-CPT-11 can specifically target EGFR-expressing cancer cells and strongly affect the drug cytotoxicity.

### In vitro cytotoxicity of free irinotecan and the antitumor effects of CPT-11-loaded DSPE-PEG_2000_ targeting EGFR liposome

By constructing the CPT-11-loaded DSPE-PEG2000 targeting EGFR liposome formulation, we hoped to improve the therapeutic relevance and effectiveness of CPT-11. We created a CPT-11-loaded DSPE-PEG2000 targeting EGFR liposome using a DSPE-PEG2000 conjugate with CPT-11 loaded into its hydrophilic core and anti-EGFR, and its targeting capability is depicted in Additional file 1: Fig. S1.

**Fig. 4:**
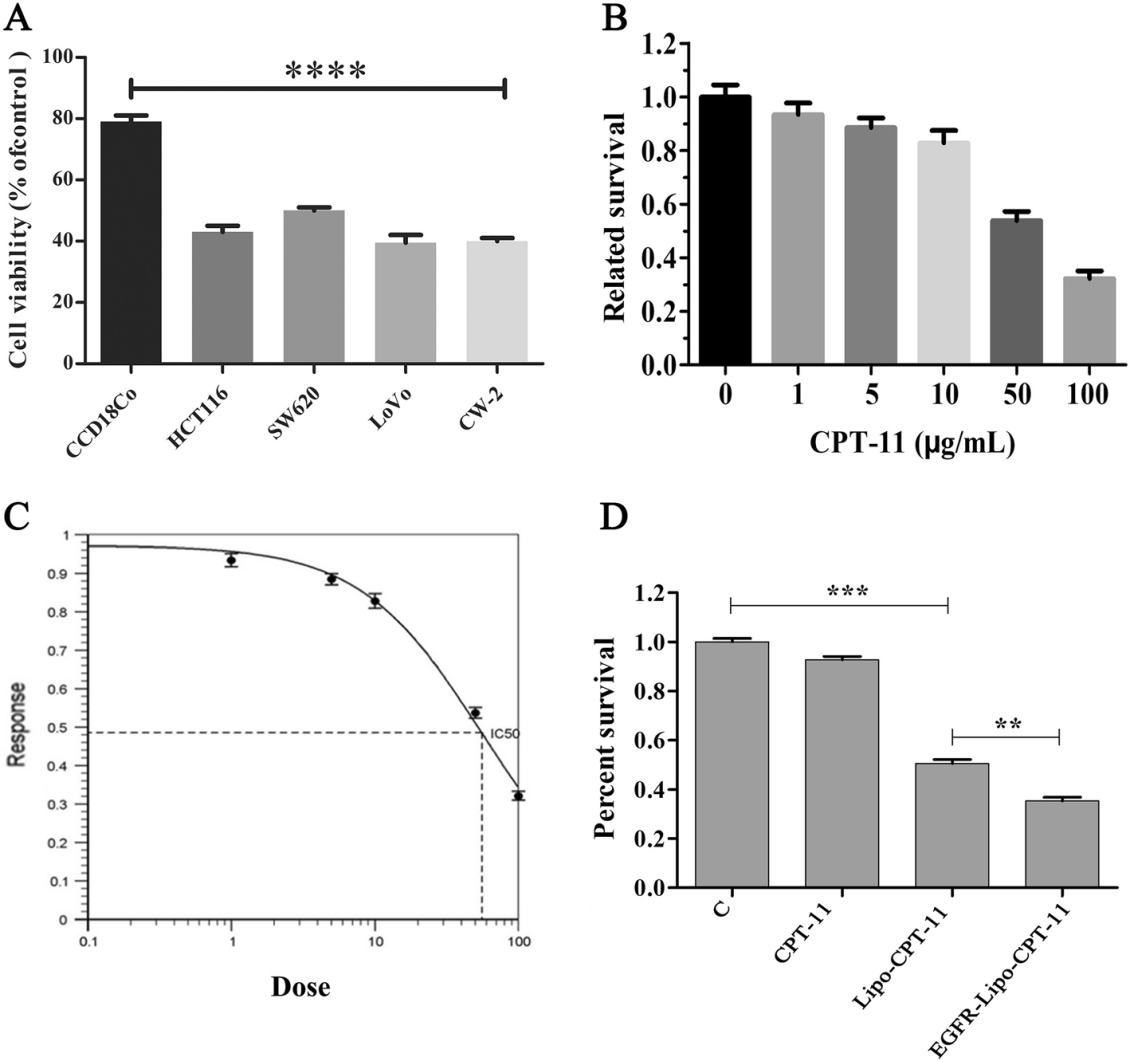
In vitro cytotoxicity of free irinotecan and the antitumor activity of CPT-11-loaded DSPE-PEG_2000_ targeting EGFR liposome. **A** Cell viability of HCT116, SW620, CW-2 and LoVo colon cancer cells using CCD18Co normal primary cells to control CRC toxicity. **B** In vitro cytotoxicity of free CPT-11 at the concentration of 1, 5, 10, 50 and 100 μg/mL; **C** the antitumor activity of CPT-11-loaded DSPE-PEG_2000_ targeting EGFR liposome in vitro; **D** survival rate of cancer cells treated with free CPT-11, CPT-11-Lipo and EGFR-Lipo-CPT-11, compared with the normal primary cells (control), generated the lowest (**p < 0.01, ***p < 0.001)

HCT116, SW620, CW-2 and LoVo colon cancer cells were cultured in 96-well plates at a density of 1 × 10^5^ cells/well for 24 h using the normal primary CCD-18Co cell lines as normal control. After adding 10 μM of CPT-11 to the medium and incubation for 72 h, we found that cytotoxic effects in CRC were significantly higher than in CCD-18Co cells normal control. At the same time, there were no significant differences among all CRC cells (Fig. 4A). The above data suggested that CPT-11 induced specific cytotoxicity CRC. SW620 cell lines were then selected to measure the cytotoxicity of CPT-11. At first, different concentrations of CPT-11, including 1, 5, 10, 50 and 100 μg/mL were used to treat SW620 cells and measure cell viability. The free CPT-11 induced dose-dependent toxicity on SW620 cells, and the IC50 of CPT-11 was 55.45 g/mL (Fig. 4B and C). Then, we evaluated the induced cell viability of SW620 high EGFR-expressing cancer cell lines under three treatments, including free CPT-11, CPT-11-loaded liposome, and CPT-11-loaded DSPE-PEG2000 targeting EGFR liposome using PBS treatment cells as a control. Compared with the control, cells treated with EGFR-Lipo-CPT-11 exhibited the lowest survival rate than the Lipo-CPT-11 and free CPT-11, respectively (Fig. 4D). These results suggested that the in vitro antitumor effects of the prepared EGFR-Lipo-CPT-11 were significantly enhanced compared with both free CPT-11 and Lipo-CPT-1.

### CPT-11-loaded DSPE-PEG 2000 targeting EGFR liposome enhanced the intracellular ROS formation

**Fig. 5:**
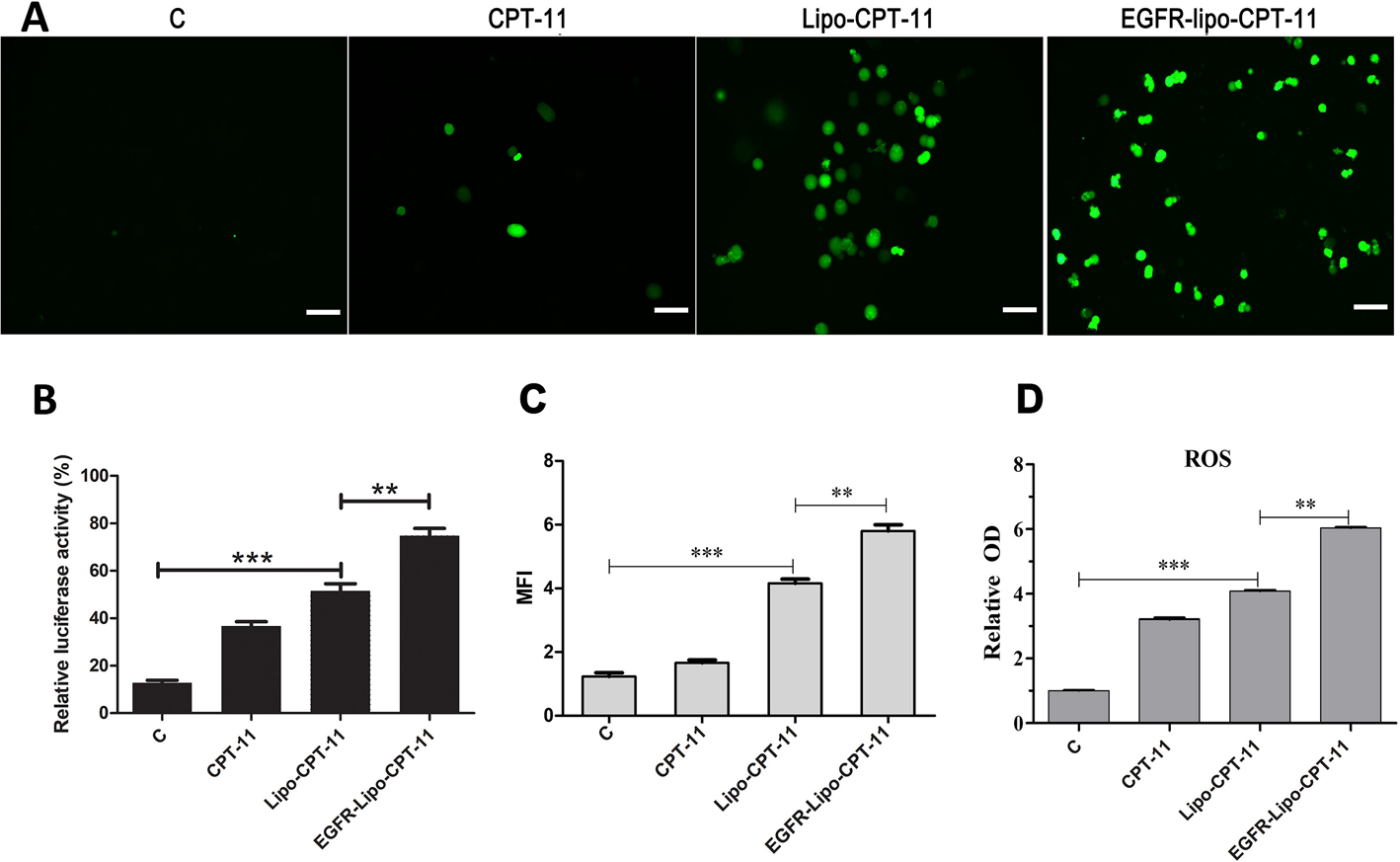
ROS formation induced by CPT-11-loaded DSPE-PEG_2000_ targeting EGFR liposome. **A** Fluorescent intensity of CPT-11, CPT-11-Lipo, EGFR-Lipo-CPT-11 and control cells. Original magnification 100×. scale bar: 100 µm. **B** The scale bar representation of the fluorescent intensity. **C** Mean fluorescent intensity (MFI) of CPT-11, CPT-11-Lipo, EGFR-Lipo-CPT-11 and control cells; **D** intracellular ROS level of CPT-11, CPT-11-Lipo, EGFR-Lipo-CPT-11 and control cells (**p < 0.01, ***p < 0.001)

High ROS levels can inhibit the drug resistance of cancer cells and promote cancer cell proliferation and metastasis in response to various chemotherapy. Here, intracellular ROS was measured in human colon cancer cells SW620 treated with CPT-11-loaded-liposome or free CPT-11 or CPT-11-loaded EGFR-targeted DSPE-PEG_2000_ liposome (EGFR-Lipo-CPT-11). The immunofluorescence assay used to visualize the intracellular ROS accumulation revealed that the intracellular ROS level generated in cells under EGFR-Lipo-CPT-11 treatment was about 1.5-fold higher than that of the free CPT-11, Lipo-CPT-11, when compared with the control (Fig. 5A and B). The highest mean fluorescence intensity (MFI) displayed by the EGFR-Lipo-CPT-11 group precisely confirmed that EGFR-Lipo-CPT-11 treatment strongly enhanced the ROS production SW620 colon cancer cell lines, besides similar results with the cell viability test (Fig. 5C). Besides, the release profile of CPT-11 from Lipo-CPT-11 and EGFR-Lipo-CPT-11 was evaluated in PBS containing 1% Triton X-100, and the OD results were similar with immunofluorescence data (Fig. 5D). These results confirmed that the CPT-11-loaded EGFR-targeted DSPE-PEG_2000_ liposome improved the production and accumulation of ROS in SW620 colon cancer cell lines.

### EGFR-Lipo-IRI treatment stimulated the caspase-3-induced apoptosis in SW620 cells in vitro

**Fig. 6:**
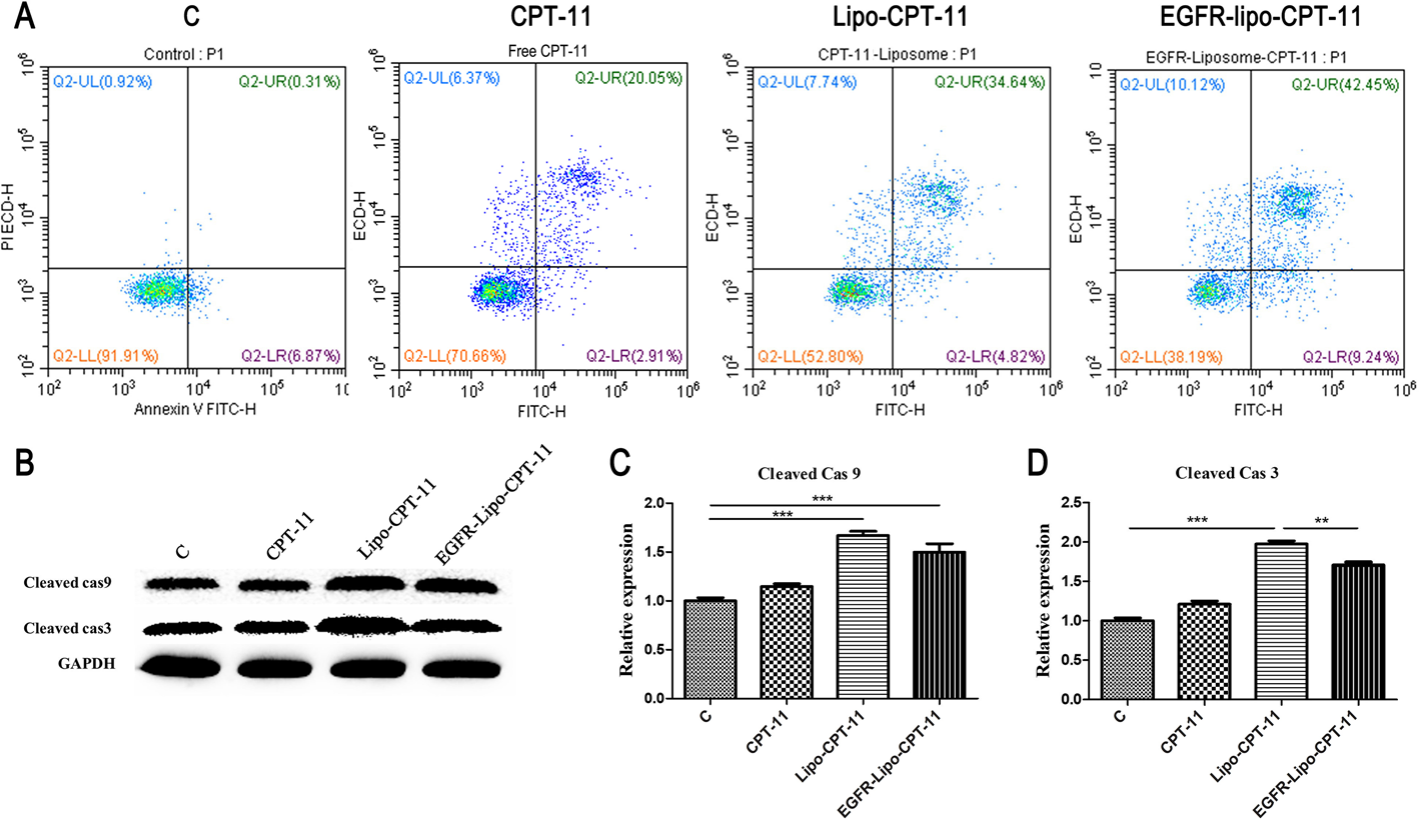
The apoptosis effect of CPT-11-loaded DSPE-PEG_2000_ targeting EGFR liposome in vitro. **A** Flow cytometry was performed to assess the apoptosis effects of CPT-11, CPT-11-Lipo, EGFR-Lipo-CPT-11, and control; **B**–**D** Western blot results showing the protein levels of cleaved-caspase-3 and cleaved-caspase-9 from four different groups mentioned above and their gray value (**p < 0.01, ***p < 0.001)

Chemotherapeutic drug CPT-11 is reputed as a pro-apoptotic agent; it was found to eliminate macrophage in CPT-11-treated mice by provoking apoptosis [19]. Flow cytometry through Annexin V/PI staining was applied to evaluate the apoptosis rate of SW620 cells treated with CPT-11-Lipo or EGFR-Lipo-CPT-11. After 24 h, and we noted 20.05%, 34.64%, and 42.45% of apoptosis rates in SW620 cells treated with free CPT-11, CPT-11-Lipo, EGFR-Lipo-CPT-11, respectively. These results confirmed that EGFR-Lipo-CPT-11 induced the strongest apoptosis rate than the CPT-11-Lipo group (Fig. 6A). Moreover, previous studies have reported the activation of caspase-3 or its related enzymes cleaved the EGFR during apoptosis [20]. Thus, the protein levels of both cleaved-caspase-3 and cleaved-caspase-9 were investigated for different treatment groups through western blot analysis. The results indicated that the protein levels of cleaved-caspase-3 and cleaved-caspase-9 were considerably upregulated in cells treated with EGFR-Lipo-CPT-11 compared with that of CPT-11-Lipo. The above results suggested that both Lipo-CPT-11 and EGFR-Lipo-CPT-11 induced massive apoptosis activity by enhancing cleavage of the critical caspase-3 protein in SW620 cells, although targeting EGFR generated a most pronounced effect (Fig. 6B).

### EGFR-Lipo-CPT-11 inhibited tumor growth in the SW620 xenograft model in vivo

**Fig. 7:**
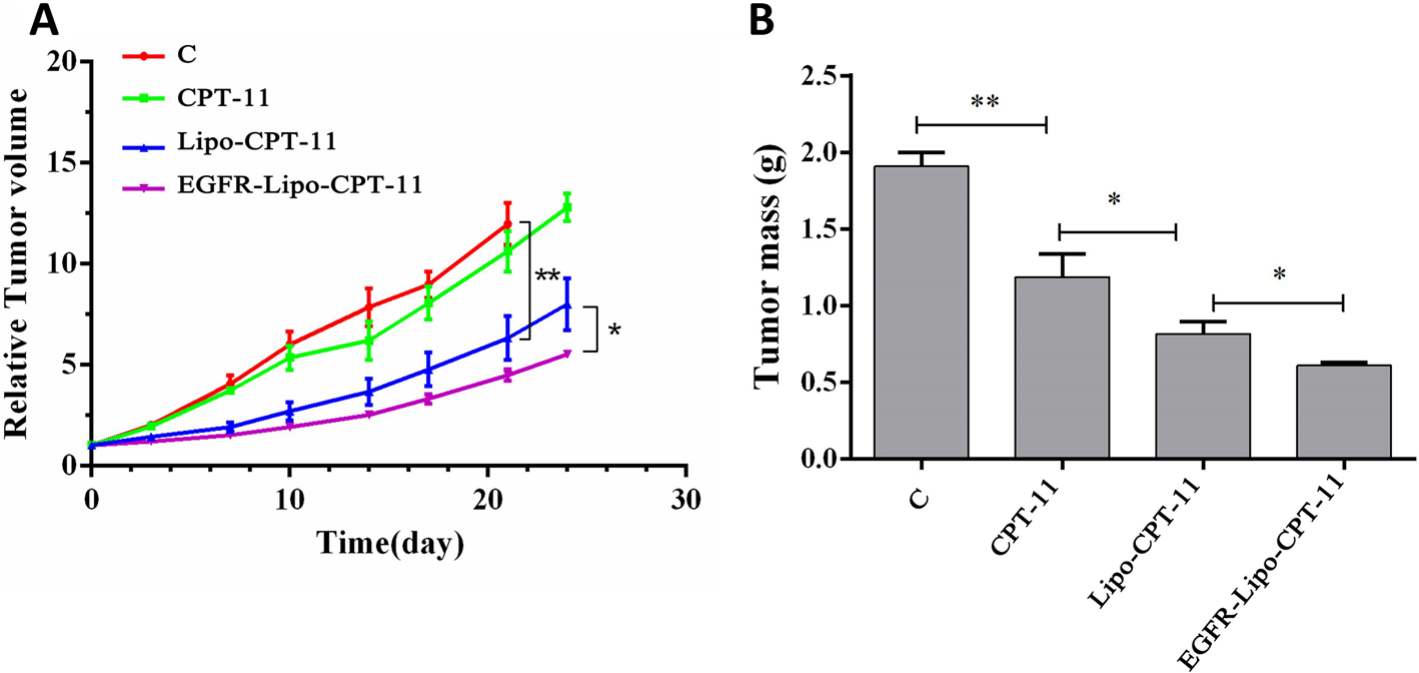
In vivo tumor inhibition effects of CPT-11-loaded DSPE-PEG_2000_ targeting EGFR liposome in the SW620 xenograft model. **A** The tumor volume of nudes treated with CPT-11, CPT-11-Lipo, EGFR-Lipo-CPT-11 and control every 3 days for 24 days; **B** the tumor mass of nudes treated with four different treatment groups mentioned above (*p < 0.05, **p < 0.01). CPT-11, irinotecan; Lipo-CPT-11, CPT-11 loaded liposome; EGFR-Lipo-CPT-11; CPT-11-loaded DSPE-PEG2000 targeting EGFR liposome

CPT-11 has been approved as first-line therapy for various advanced stages (metastatic phase) of cancers like colon and gastric cancers. Previous studies have demonstrated that administration of CPT-11 strongly reduced the growth of glioma tumors by blocking angiogenesis, reducing the number of tumor vessels and the surface of hypoxic lesions, and significantly decreased expression of VEGF [21]. To further investigate the effect of combining EGFR-Lipo-CPT-11 treatment in colon cancer cells, mice were intravenously injected with PBS (Control), free CPT-11, or Lipo-CPT-11 or EGFR-Lipo-CPT-11. Then, the tumor growth inhibition was assessed in SW620 cells xenografts, tumor model. From the tumor growth curve, the free CPT-11 treatment induced little inhibitory effect on the tumor growth. On the other hand, after loading, the antitumor effects of CPT-11-loaded DSPE-PEG2000 targeting EGFR liposome became significantly higher than that of CPT-11-loaded liposome (Fig. 7A and B). These results indicate that the CPT-11-loaded DSPE-PEG2000 targeting EGFR liposome enhanced the antitumor effects of CPT-11 through the direct target of EGFR on the cell surface membrane.

## Discussion

Nowadays, irinotecan (CPT-11) is widely used as the main chemotherapeutic agent for gastrointestinal cancers like pancreatic ductal adenocarcinoma and colon cancer [22]. Also, metastatic colorectal cancer patients are generally treated by a combination of CPT-11 and fluorouracil or leucovorin [23] or administering a higher dose of CPT-11 to patients who developed an intolerance to fluorouracil [24]. However, the clinical use of CPT-11 is majorly limited by their severe side effects like neutropenia and diarrhea. Many researchers are motivated to find a better drug delivery system to reduce important chemotherapies’ side effects. In this regard, nanocarriers like liposomes and polymeric nano-conjugates are the most recommended [25]. The liposome represents a natural lipid-based nanoparticle that is used to enhance the antitumor activity of low molecular weight drugs by extending its biological half-life and relieving its systemic toxicity [26]. However, the prepared liposomes should have a controlled size and surface decorated with target-specific antibodies [27]. For example, the tyrosine-modified irinotecan-loaded liposomes targeting LAT1 and ATB^0,+^ showed good prospects for tumor therapy and industrial production [28]. Liposomes have been widely described as efficient drug delivery systems in many studies. The range of 40 to 100 nm of liposome diameters was defined as suitable for the maximum drug infusion in both rodent and primate brains by convection-enhanced delivery CED [29]. The first combined agent nano-liposomal CPT-11, showed that stable encapsulation of CPT-11 in lipidic nanoparticles improved the CPT-11 diffusion and its anticancer activities in brain cancers. In this study, we used the self-assembly method to investigate the effect of CPT-11-loaded DSPE-PEG_2000_ targeting EGFR liposome on the SW620 colon cancer cells. This method offers simple and large-scale production, safe and stable prepared drug-loaded liposome DSPE-PEG_2000,_ for drug safety by the use of lipid-based nanocarriers [30–32]. The transmission electron microscopy (TEM) results showed that before and after loading, the average sizes of liposomal nanoparticles increased from 84.6 ± 1.2 nm to 150.4 nm ± 0.8, indicating that liposomal nanocarriers successfully loaded the irinotecan and EGFR (Fig. 1). This justified the stability and the successful assembly of the novel CPT-11 delivery system, since phagocytosis can involve molecules larger 150 nm of diameter liposomes. Moreover, the average size of EGFR-Lipo-CPT-11 was unchanged during the whole observation period, demonstrating good physical stability of the EGFR-Lipo-CPT-11. Interestingly, the in vitro release rate of irinotecan from EGFR-Lipo-CPT-11 displayed a rapid and sustained elimination of CPT-11, suggesting that the CPT-11-release in the cytoplasm could be strongly enhanced by liposome encapsulation method (Fig. 2). In addition, the cytotoxicity of EGFR-Lipo-CPT-11 and Lipo-CPT-11 were associated with the expression levels of EGFR in CRC cells. EGFR-Lipo-CPT-11 induced weaker cell viability than the free CPT-11, suggesting that the PEG-loaded irinotecan are slowly absorbed by cells because of the undergoing drug-release process, while the free CPT-11 was rapidly transiting into the cells through passive diffusion. However, EGFR-Lipo-CPT-11 exhibited the strongest effect in SW620 high EGFR-expressed cell lines, showing that the PEG-loaded CPT-11 specifically targets EGFR-expressing cancer cells (Fig. 3).

DSPE-PEG_2000_ curcumin-loaded liposome improves the antitumor activity of pancreatic adenocarcinoma cell lines AsPC-1 and BxPC-3. Mahmud et al*.* suggested that DSPE-PEG_2000_ modified the surface of the liposome and prolonged circulation time and increased the accumulation of liposomes in tumor tissues [33]. As FDA approved the application of DSPE-PEG2000 (1,2-distearoyl-sn-glycero-3-phosphoethanolamine-PEG2000) in the drug formulations [34] and knowing the importance of EGFR in the treatment of colorectal cancers (CRCs) [10, 11], we sought to evaluate the antitumor effects of EGFR-Lipo-CPT-11 through cell proliferation, ROS formation, and cell apoptosis in vitro. As a result, the CPT-11-loaded DSPE-PEG_2000_ targeting EGFR liposome greatly enhanced the antitumor effects of CPT-11-loaded liposome in CRC (Fig. 4). Also, the intracellular ROS induced by the prepared EGFR-Lipo-CPT-11 was significantly increased compared with that of free irinotecan (Fig. 5). Overall, these results showed that using EGFR-targeted DSPE-PEG2000 liposomes as CPT-11 delivery system conferred lower cell viability rate and extended ROS production to CRCs. Therefore, the EGFR-target liposome delivery system could enhance the CPT-11 release in the cytoplasm and improve their antitumor activities [35, 36]. The flow cytometry assay, which detects early cell apoptosis, indicated the highest apoptotic effects in EGFR-Lipo-CPT-11 groups. CPT-11 is known to function through caspase-3-mediated apoptosis [37, 38], and the protein expression of cleaved-caspase-3 and cleaved-caspase-9 were strongly elevated in cells treated with EGFR-Lipo-CPT-11 (Fig. 6). Thus, using EGFR-targeted DSPE-PEG2000 liposomes as CPT-11 delivery system could improve the therapeutic efficacy of CPT-11 by enhancing the caspase-mediated apoptosis pathway in tumor cells. Finally, the in vivo antitumor effect of EGFR-Lipo-CPT-11 was confirmed through the xenograft model. The inhibition tumor growth of the prepared CPT-11-loaded liposome was better than that of free CPT-11 by the related tumor volume and weight (Fig. 7). The inhibition of the tumor growth and the reduction of its related volume highlighted the fact that the EGFR-target liposome delivery system optimized the pharmacodynamics and pharmacokinetics of the CPT-11 drugs in CRCs.

## Conclusion

Overall, CPT-11-loaded DSPE-PEG_2000_ targeting EGFR liposome prepared in this study represents an accurate carrier for irinotecan delivery and exhibits great clinical application potential. The functional characterization of the CPT-11-loaded DSPE-PEG_2000_ targeting EGFR liposome was successfully carried out. The typical spherical or irregular shape of prepared liposomes with good physical stability and enough drug loading ensured the therapy efficacy. Our results from both in vitro and in vivo experiments demonstrated that the prepared liposome produced better antitumor activity compared to free CPT-11, an extended period outflow of irinotecan took time from PEGylated liposomes and proposed encouraging expectations on the use of EGFR-targeted DSPE-PEG2000 liposomes as CPT-11 delivery system for CRC targeting therapy.

## Materials and methods

### Materials

Human colon cancer cell line: SW620, CW-2, LoVo and HCT116 purchased from ATCC (90% DMEM + 10% FBS) 0.1,2-distearoyl-sn-glycero-3-phosphoethanolamine-*N*-[methoxy(polyethyleneglycol)-2000(ammonium salt) (DSPE-PEG2000-NH2) were purchased from Ananti Polar Lipids, Inc. (Beijing, China). Cetuximab, a monoclonal antibody anti-EGFR, was purchased from Shanghai TheraMabsBiotech (product number TM-Beva-00002). DMSO (D8371, Solarbio), dialysis bag (3.5KD, F132590, BBI), DMEM medium (Cat. No. 11965-126), fetal bovine serum (FBS) (product number 16000-044), trypsin and double antibodies were purchased from Gibco company. MTT powder was purchased from Azerbaijan Latin (Cat. No. T100896-1g), irinotecan (Irinotecan, CPT-11), purchased from Solarbio (SI8480-20 mg), EDC purchased from Sigma (D8740-500 mg), NHS purchased from Sigma (D8740-500 mg) Balb/c nude mice were purchased from Weitong Lihua.

Main instruments: ordinary microscope (OLYMPUS company in Japan), small desktop refrigerated centrifuge (Technology University Zhongjia), cell incubator (American Thermoelectric), heating magnetic stirrer (C-MAG HS 10, IKA) CM-H_2_DCFDA were obtained from ThermoFisher.

### Cell and cell culture

CCD-18Co primary colon cell lines and HCT116, SW620, CW-2 and LoVo colon cancer cells and were all purchased from American Type Culture Collection (ATCC, Rockville, MD, USA) and cultured in DMEM supplemented with 10% FBS (No-16000-044/Gibco, USA) in the humid chamber, maintaining 37 °C and 5% CO_2_. The concentrations of 1, 5, 10, 50, and 100 of μg/mL CPT-11 (SI8480-20 mg; Solarbio) were, respectively, added into the cell’s medium for 48 h.

### Animal experiments

In vivo experiments were performed using female BALB/c nude mice (4–6 weeks old) obtained from Shanghai SLAC laboratory animal Co., Ltd (Shanghai, China). The whole animal experiment was conducted following the Animal Care Guidelines of Rongchang District People’s Hospital of Chongqing. All the nudes were housed, fed with a regular diet, given acidified water without antibiotics. We injected subcutaneously with 100 μL SW620 cell suspensions (1 × 10^6^ cells/mL) into the right flank after 1 week. SW620 were cultured, digested, centrifuged, collected, and diluted to 5 * 107/mL; then, the suspension was subcutaneously injected on the right limbs of 6-week-old mice morphologically similar (1 week in advance). When the tumor formation was observed in all mice after 2 weeks of inoculation, the mice were randomly divided into four groups, with three mice in each group, including controlling groups treated with PBS (C), groups treated with CPT-11-loaded liposome (Lipo-CPT-11), free CPT-11 (CPT-11), and CPT-11-loaded EGFR-targeted DSPE-PEG_2000_ liposome (EGFR-Lipo-CPT-11). When the tumor volume increased to about 100 mm^3^, the mice were injected intravenously once a week for 3 weeks. The tumor volumes were measured every 3–4 days; then, after 4 weeks of treatment, the mice were quickly dislocated and killed to collect and weigh the tumor. The tumor volume (*V*) was calculated as follows: *V* = (*L* × *W*^2^)/2, where *L* and *W* were the longest and the shortest diameter of the tumor.

### Preparation of CPT-11-loaded DSPE-PEG_2000_ targeting EGFR liposome

2 mg of the DSPE-PEG2000-NH2 purchased from Ananti Polar Lipids, Inc. (Beijing, China) was dissolved into 1 mL of DMSO (D8371, Solarbio), to obtain 2 mg/mL solution A, and 2 mg of irinotecan (SI8480-20 mg, Solarbio) was dissolved into 1 mL of DMSO to form 2 mg/mL solution B. solutions A and B were well mixed according to the 1:1 mass ratio and slowly stirred at room temperature for 2 h in the dark. Then, 2 mL of the mixed solution was transferred to a dialysis bag (3.5KD, F132590, BBI) to perform overnight dialysis in 18.2 MΩ deionized water and 2.62 mL of CPT-11-loaded DSPE-PEG2000 liposome (Lipo-CPT-11). After the characterization of particle size and the stability of the loaded liposome nanoparticles, the coupling of EGFRs to CPT-11-loaded DSPE-PEG2000 liposomes were prepared using EDC and NHS as coupling reagents. Briefly, 200 μL of CPT-11-loaded DSPE-PEG2000 liposome suspension in PBS buffer were added to a prepared solution of 100 μL of 0.25 M EDC and 100 μL of 0.25 M NHS, and the mixture was incubated for 10 min at room temperature with a pH adjusted to 7.5 using 1 M NaOH. Then, 20 μL of antibody anti-EGFR (product number TM-Beva-00002) (100 mg/mL) was added to the mixture, and the solution was gently stirred for 8 h at 4 °C. Finally, EGFR-coated CPT-11-loaded DSPE-PEG2000 liposomes (EGFR-Lipo-CPT-11) were separated from unbound antibody anti-EGFR using a saline pre-equilibrated Sepharose CL-4B column, and the upper solution containing EGFR-coated CPT-11-loaded DSPE-PEG2000 liposomes was collected, pooled, sterilized and stored under nitrogen at 4 °C.

### The morphology and particle size of CPT-11-loaded DSPE-PEG_2000_ liposome

The shapes and surface morphologies of CPT-11-loaded EGFR-targeted DSPE-PEG_2000_ liposomes were observed via a transmission electron microscope (H-6009IV, Hitachi, Japan). The particle sizes of these liposomes were also determined by dynamic light scattering (Malvern Nano-ZS 90) at room temperature, and all measurements were repeated three times. To quantify the EGFR overexpressed tumor, western blot analysis was applied on four different CRC cell lines, including CW-2, LoVo, SW620 and HCT116 cells. Briefly, All the specimens were heated in sample buffer and sorted using SDS-PAGE with 10% gradient. The recovered proteins were then moved to a PVDF membrane (Millipore, Bedford, MA, USA) blocked in a 5% skim milk solution (Becton–Dickinson & Co., Sparks, MD, USA). After 2 h in blocking buffer (TBS containing 5% skim milk and 0.1% Tween20), the PVDF membrane was incubated with the primary antibody. Then, anti-IgG at 1:1500 was used to detect the specific secondary antibody, and the chemiluminescence system (ECL, Pierce) was used to identify relative proteins.

### The physical stability, drug loading, and cumulative release profile in vitro of CPT-11-loaded DSPE-PEG_2000_ targeting EGFR liposome

The physical stability of liposomes was primarily determined by the critical micelle concentration in an aqueous solution at 25 °C. Micellar diameter changes as a function of time and scattering intensities were evaluated by DLS as mentioned above. 10 mg of CPT-11-loaded DSPE-PEG2000 targeting EGFR liposome was dissolved into 0.1 mL acetonitrile, and the amount of irinotecan was measured by HPLC. The drug loading was calculated as follows: drug/(polymer + drug) × 100%. The lyophilized powder of CPT-11-loaded DSPE-PEG2000 targeting EGFR liposome was dissolved into the deionized water, and 5 mL of them were put into a dialysis bag (molecular weight cut-off 3500 g/mol). Then, the bag was immersed in 30 mL of phosphate-buffered saline (PBS, pH 7.4) containing Tween-80 (0.5% w/w), and the medium was stirred at 70 rpm at 37 °C. Samples were collected at 6th, 12th, 24th, 36th h, 48th h, and 72nd h, and the same volume of fresh PBS was added to maintain the buffer volume unchanged. The concentration of the released irinotecan in the dialysis media was determined by HPLC (LC-10ATvp, Shimadzu) with a C18 column (Symmetry shield TM RP18, 3.9 mm × 150 mm, from Waters) at 25 °C. The standard curve was evaluated at 10, 25, 50, 100, 150, and 200 ug/mL, while the drug concentrations were calculated at each time point. Next, the accumulative release amount of irinotecan was calculated using a calibration curve and expressed as the Percentage of released concentration through the following formulation: Cumulative percentage release (%) = Volume of sample withdrawn (mL)/bath volume (*v*) × *P* (*t* − 1) + Pt. Where Pt = Percentage released at time *t* and *P* (*t* − 1) = Percentage release before ‘*t*’.

### In vitro cytotoxicity of free CPT-11 and the antitumor activity of CPT-11-loaded DSPE-PEG_2000_ targeting EGFR liposome

SW620 cells were exposed to the different concentrations of irinotecan (1, 5, 10, 50, and 100 μg/mL), and cell viability was measured to explore the IC50 of irinotecan through MTT assay. Then, cells were separated into four groups, including the control group (C), cells treated with free CPT-11 (CPT-11), CPT-11 loaded liposome (Lipo-CPT-11), and CPT-11-loaded DSPE-PEG_2000_ targeting EGFR liposome (EGFR-Lipo-CPT-11), respectively. Then, MTT assays were performed to evaluate the antitumor activities produced by the four groups.

### MTT assay

According to the grouping, control, EGFR liposome, CPT-11-loaded DSPE-PEG2000 particles and CPT-11-loaded DSPE-PEG2000 targeting EGFR liposome groups were cultured in a 37 °C, 5% CO_2_ saturated humidity incubator for 48 h. Add 20 µL MTT solution (Sigma-Aldrich, MO, USA) to each well and continue to incubate for 4 h, discard the supernatant, add 150 µL DMSO to each well and shake for 15 min to dissolve the whole crystals. An enzyme-linked immunoassay detected the absorbance value (A570) of each well at 570 nm, and the growth inhibition rate of each group of cells was calculated. The calculation formula was applied as follows: Cell viability (%) = (experimental group absorbance value/control group absorbance value) × 100%.

### Reactive oxygen species (ROS) analysis in vitro

Reactive oxygen species from cellular was determined using the conversion of non-fluorescent 5, 6-Chloromethyl-2V, 7V dichloro dihydro fluorescein diacetate (CM-H_2_DCFDA) to its fluorescent derivative (DCF) by Reactive Oxygen Species Assay Kit (Abcam, USA) according to the manufacturer’s recommendation. Four groups of SW620 cells, respectively, treated with free CPT-11, CPT-11-loaded DSPE-PEG2000 particles, CPT-11-loaded DSPE-PEG 2000 targeting EGFR liposome, and the control group. Then, 50 μmol/L DCFH2-DA were added for 30 min, washed with PBS, and centrifuged. Furthermore, PBS containing 1% Triton X-100 was added, and the intensity of DCF fluorescence (OD) was assessed via a microplate reader at 485 nm of excitation and 530 nm of emission.

### Cell apoptosis assay

The SW620 cells from different treatment groups were collected by centrifugation at 1500 rpm and were stained with Annexin V-FITC for 30 min and propidium iodide (PI) for 5 min after being washed with pre-cooling PBS. Finally, the samples were subjected to a fluorescence-activated cell-sorting (FACS) flow cytometer (BD Biosciences, San Jose, CA, USA). Flow cytometry was performed to measure cell apoptosis.

### Western blot analysis

Total protein was extracted from lysed cells, and the equivalent amount of protein was separated with 10% SDS-PAGE, then transfected to a PVDF membrane (Millipore). After being blocked with 5% nonfat milk (Cell Signaling Technology), the membranes were incubated with primary antibodies against cleaved-caspase-9 (Abcam, ab2324) and cleaved-caspase-3 (Abcam, ab208003) and GAPDH at 4 °C overnight followed by secondary antibody incubation. The protein bands were visualized using CLARITYTM Western ECL substrate (Bio-Rad), and the protein level was quantified and normalized with GAPDH.

### Statistical analysis

SPSS version 18.0 was used for statistical analysis through the one-way ANOVA method. All values were presented as the mean ± SD differences with *p* < 0.05 were considered statistically significant.

## Supplementary Information

**Additional file 1: Figure S1.** Schematic illustration of CPT-11-loaded DSPE-PEG2000 targeting EGFR liposome formulation. Abbreviations: CPT-11, Irinotecan; DSPE-PEG 2000, 1,2-distearoyl-sn-glycero-3-phosphoethanolamine-*N*-[methoxy(polyethylene glycol)-2000]; EGFR, Epidermal Growth Factor Receptor.

## Data Availability

The datasets used and/or analyzed during the current study are available from the corresponding author on reasonable request.

## Abbreviations

CRC: Colorectal cancer
CPT-11: Camptothecin-11 (irinotecan)
DSPE-PEG2000: 1,2-Distearoyl-sn-glycero-3-phosphoethanolamine-*N*-[amino (polyethylene glycol)-2000] (ammonium salt)
EGFR: Epidermal growth factor receptor
EGFR-Lipo-CPT-11 or EGFR-Lipo-IRI: Irinotecan-loaded EGFR-targeted DSPE-PEG2000 liposome
Lipo-CPT-11: CPT-11-loaded DSPE-PEG2000 liposome
ROS: Reactive oxygen species
SW620: Human metastatic colorectal adenocarcinoma cells
EPR: Enhanced permeability and retention
PD-L1: Programmed death ligand-1
IC50: Concentration at which a substance exerts half of its maximal inhibitory effect
5-FU: 5-Fluoroacyl
VLF: Venlafaxine
MTT: 3-(4,5-Dimethylthiazol-2-yl)-2,5-diphenyl tetrazolium bromide
DNA: Deoxyribonucleic acid
RNA: Ribonucleic acid
