# Supplementary figures and images for: Targeted delivery of irinotecan to colon cancer cells using epidermal growth factor receptor-conjugated liposomes
Source: Biomed Eng Online. 2022 Aug 2;21:53. doi: 10.1186/s12938-022-01012-8 (PMC9344698; doi:10.1186/s12938-022-01012-8)

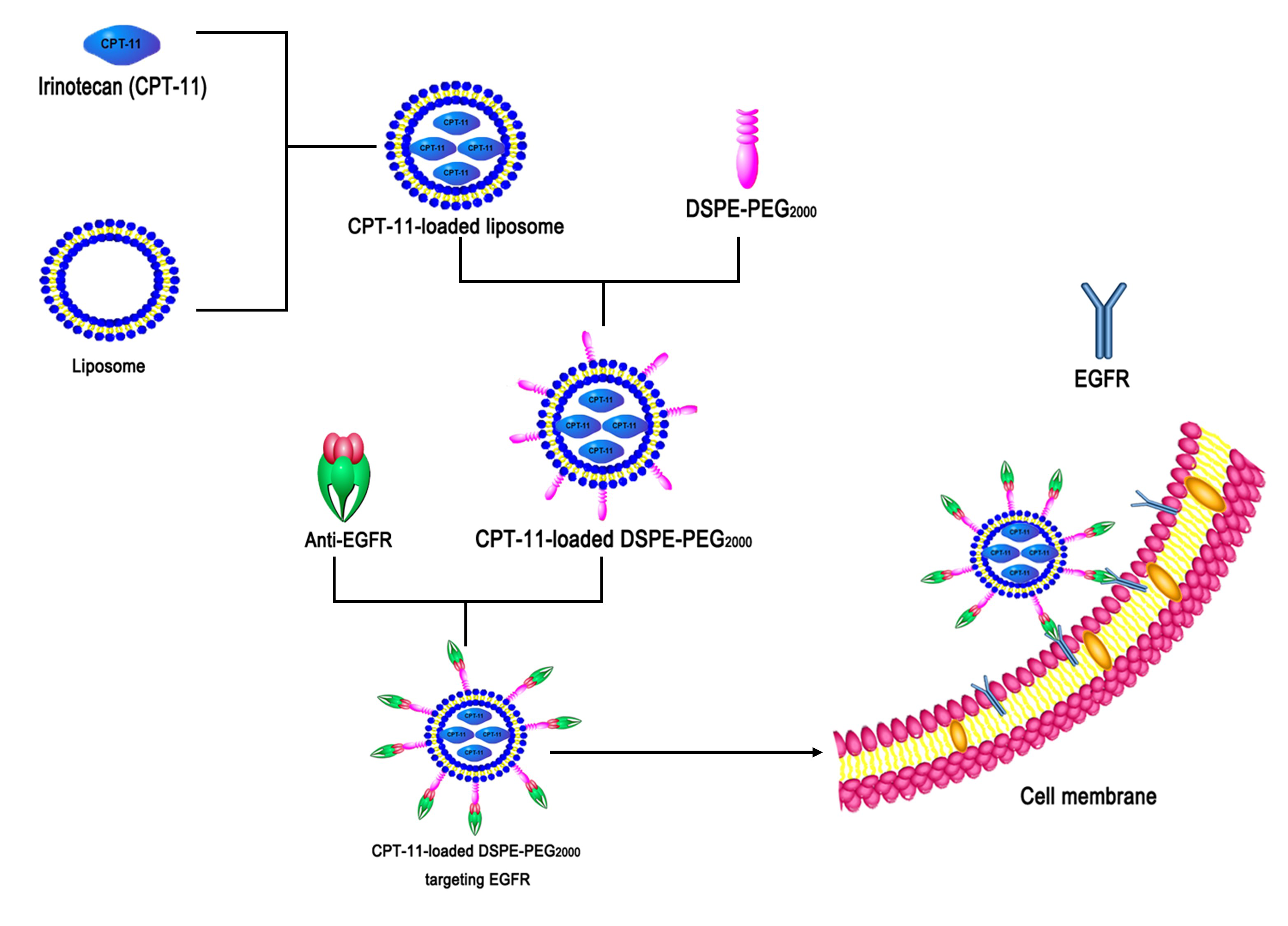

Supplement: Supplementary file 1 — Additional file 1: Figure S1. Schematic illustration of CPT-11-loaded DSPE-PEG2000 targeting EGFR liposome formulation. Abbreviations: CPT-11, Irinotecan; DSPE-PEG 2000, 1,2-distearoyl-sn-glycero-3-phosphoethanolamine-N-[methoxy(polyethylene glycol)-2000]; EGFR, Epidermal Growth Factor Receptor. [file 12938_2022_1012_MOESM1_ESM.tif]
